# Operationalizing a 3-year standalone, accelerated medical school curriculum to nurture physicians to become primary care and health system leaders

**DOI:** 10.1080/10872981.2024.2367821

**Published:** 2024-06-19

**Authors:** Maria Lyn Quintos-Alagheband, Orla O’Donoghue, Gladys M. Ayala, Steven Carsons, Nobuyuki Miyawaki, Arsenia Asuncion, Francis Faustino, Patricia Janicke, Jeffrey Berger, Dana Ribeiro Miller, Clothilde Castiglia, Isabella Harnick, Steven Shelov

**Affiliations:** a Department of Pediatrics, New York University Grossman Long Island School of Medicine, Mineola, NY, USA; b Department of Foundations of Medicine, New York University Grossman Long Island School of Medicine, Mineola, NY, USA; c Department of Internal Medicine, New York University Grossman Long Island School of Medicine, Mineola, NY, USA; d Department of Family Medicine, New York University Grossman Long Island School of Medicine, Mineola, NY, USA; e NYU Langone Health, New York University Grossman Long Island School of Medicine, Mineola, NY, USA

**Keywords:** Primary care, accelerated medical school pathway, health systems science, problem based learning, continuous ambulatory practice experience, social science, humanities, curriculum development, undergraduate medical education, longitudinal integration

## Abstract

The United States faces a shortage of primary care physicians. To address this, there have been pioneering efforts to develop accelerated pathways with a primary care focused curriculum for undergraduate medical education. The New York University Grossman Long Island School of Medicine (NYU GLISOM) was conceptualized as the first standalone, accelerated, tuition-free program in the US in over 100 years, with mission-centered curriculum on primary care and health system leadership. The aim of this article is to map the process for the development of a three-year integrated curriculum, describe the pedagogical approach that guided the design of the longitudinal courses, share the student and faculty’s perspective about the curriculum, and describe the early outcomes of the first two graduate classes. A major key driver for curricular design is integrating longitudinal courses of Clinical Ambulatory Practice Experience (CAPE), Health Systems Science (HSS), and Learning Community - Social Sciences, Humanities, Ethics and Professionalism (LC-SHEP) over three years and active learning through Problem Based Learning (PBL). We have successfully operationalized an accelerated, standalone, integrated medical school curriculum mission-centered on primary care and health system leadership. Our outcomes reveal a higher percentage (76% N =45) of NYU GLISOM students entering primary care compared to national benchmarks. The integration of the longitudinal courses of HSS, LC-SHEP, and CAPE is a key pillar to reinforce the tenants of primary care and health system leadership. Focused interview of graduates from the pioneer cohort consistently stated that the longitudinal courses prepared them well for residency in primary care and as a health systems’ change agent. Despite the challenges of an accelerated program, NYU GLISOM successfully integrated the longitudinal courses with optimal performance and achievement of educational program objectives. Our experience can serve as a model for innovation and design of an accelerated three-year primary care curriculum.

## Background

Numerous data points support that the United States will see an increasing shortage of physicians for the next decade. In 2020, the American Association of Medical Colleges (AAMC) estimated a shortage of 124,000 physicians by 2034 [1]. Although this shortage will affect both primary care and subspecialty need; the bigger crisis will be in primary care with projected physician demand for primary care in 2034 as 47,300 in the Northeast and 287,000 in all of the United States [2]. The reduced supply of US medical graduates entering primary care tracks, in addition to an ageing population harboring an increased burden of chronic illnesses will exacerbate this supply and demand imbalance creating a public health crisis [3,4].

In a commentary on *Health Care, 2030: The Coming Transformation*, the authors discussed major shifts in the design of health systems and healthcare, propelled by digital health, growing consumerism, mounting financial constraints, and accelerated by Covid-19. The authors also identified gaps and barriers in the current design of health systems and needed escalation of transformation, including transition from hospital-based system to a primary care, community, and social care-based system [5]. The Institute for Healthcare Improvement calls for an urgent need to transform the prevailing systems of healthcare management and education in the United States since 2001 [6]. This parallel transformation poses both quantity and quality challenges to our medical education system and highlights the need to innovate curricula.

Literature supports the link between the availability of primary care physicians and positive patient outcomes. Starfield et al. showed that primary care improves population health and lowers health-care expenditures. They explained that ‘primary care (in contrast to specialty care) is associated with a more equitable distribution of health in populations, a finding that holds in both cross-national and within-national studies.’ [7]. Similarly, Pilkerton et al. found that ‘a greater supply of primary care physicians per capita is associated with improved cardiovascular health, lower mortality, increased lifespan, and a reduction in low birth-weight rates.’ [8]. Medical students choosing primary care careers can improve access by practicing in underserved communities, reduce healthcare costs through preventative care, bridge care coordination, and provide a holistic approach to healthcare. Primary care is the foundation of a well-functioning healthcare system and its importance in delivering high-quality care to patients cannot be overstated. One way medical schools can address the primary care physician deficiency is by influencing students’ career preferences via an efficient, engaging, and comprehensive immersion in the principles of primary care. Thus, the need to establish a primary care mission driven school is critical.

Rising medical student debt has exacerbated the primary care physician shortage by negatively impacting students that choose primary care as a specialty showed an ‘inverse relationship between the level of total educational debt and the intention to enter primary care’ [9,10]. In the 2009 AAMC Medical School Graduation Questionnaire (*n* = 9,300), medical students responded that debt had a 24% moderate to strong influence on specialty choice, 16% of which said it had a strong influence [11]. Therefore, there have been efforts by pioneering schools to develop accelerated pathways to reduce the burden of student debt and expedite entry into the workforce [12–17]. By reducing medical school to 3 years, it would increase the supply of physicians, particularly for primary care physicians and reduce the cost of medical training, without compromising clinical care [18].

To address the national shortage of primary care physicians, the NYU Langone Health (NYULH) System under the leadership of Dean and Chief Executive Officer Robert I. Grossman, MD and Vice Dean for Education Steven B. Abramson, MD committed to developing a second tuition-free medical school with an accelerated pathway, focused on primary care. The NYU Grossman Long Island School of Medicine (NYU GLISOM) was envisioned as the first standalone 3-year medical school in the US in over 100 years [14]. The faculty and leadership of NYU GLISOM operationalized a curriculum centered on the school’s mission ‘to develop preeminent physician leaders and a diverse workforce through scholarship and innovative education design anchored by the principles of primary care and health systems science.’

Our school selects applicants for admission who demonstrate personal attributes of motivation, adaptability, and resilience with a capacity for improvement to thrive in a 3-year accelerated program. To further the mission of the school for primary care, we look for applicants with significant experience in working in primary care settings or with an economically and/or socially disadvantaged population. Another aspect of the mission of the school is to produce primary care physicians who are prepared to become leaders of their community and the nation’s healthcare system, as evidenced by their prior experience in public health, community outreach, or healthcare administration.

The aim of this article is to map the process for the development of a 3-year integrated curriculum, describe the pedagogical approach that guided the design of the longitudinal courses, share the student and faculty’s perspective about the curriculum, and describe the early outcomes of the first two graduate classes.

## Design

**Figure 1.**
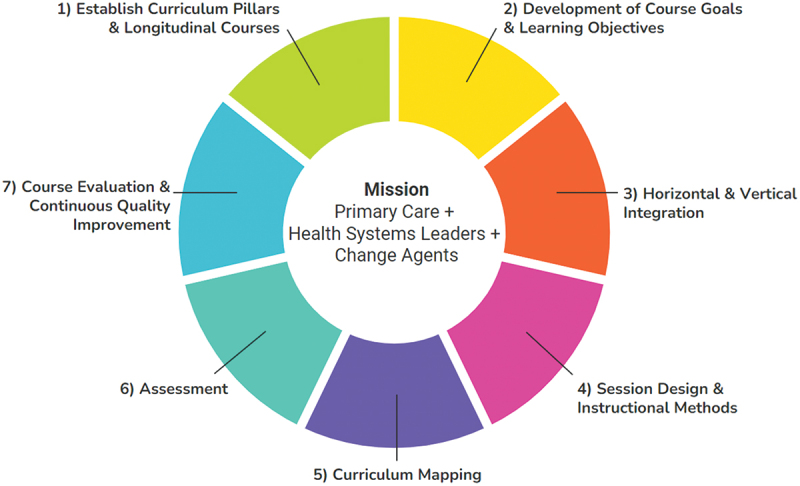
Process map for curriculum development.

The curriculum team mapped the process for the design and implementation of a 3-year, integrated medical school curriculum (Figure 1).

The integrated longitudinal courses and PBL course directors were asked to define the pedagogical approach, key drivers, and challenges and opportunities for curriculum development.

Four graduates from the pioneer class were interviewed six to eight months after their graduation. To mitigate bias, a project manager, who is part of the health system but has no affiliation with the medical school, was tasked with conducting interviews. The project manager conducted one-to-one interviews of graduates during their first year of residency in each of the four primary care tracks: 1) Internal Medicine, 2) Obstetrics & Gynecology, 3) Pediatrics, and 4) Family Medicine. Through a faculty multi-voting strategy, two graduates were selected from residency within our health system and two from external programs.

Outcome measures for the first two graduating classes were analyzed: Residency match results, Residency Readiness Surveys (RRS), Graduate Questionnaire (GQ) residency program director internship reports, United States Medical Licensing Examination (USMLE) and National Board of Medical Examiners (NBME) Health Systems Science (HSS) examinations.

In addition, a Strengths, Weaknesses, Opportunities and Threats (SWOT) analysis was completed with the course directors, representatives from the faculty team and the office of medical education.

All data was reviewed and approved by the NYU GLISOM Medical Education Research and Scholarship Committee with IRB Data Registry approval.

## Process map for curriculum development

### Operationalizing the mission and governance

A curriculum taskforce led by the founding Dean, curricular Deans, course directors, leads for assessment and evaluations, innovation, and project management, was established to develop a curriculum centered on the mission of developing primary care physicians and health system leaders. The initial curriculum taskforce had a total of 47 members; 70% are faculty from the emerging NYU GLISOM and 30% from the curriculum leadership team and representative Course Directors from the existing NYU Grossman School of Medicine faculty in Manhattan. Members from NYU GLISOM were selected from faculty who held leadership positions in teaching basic and clinical science in the legacy academic campus hospital. The task force met monthly until the formulization of the core curriculum committee exclusive to the newly founded NYU GLISOM.

The curriculum taskforce researched materials related to existing medical schools with embedded accelerated 3-year pathway Undergraduate Medical Education (UME) programs and a primary care focused curriculum [19–21]. The taskforce also reviewed Liaison Committee on Medical Education (LCME) guidelines on the ‘Structure and Function of a Medical School’ as well as the ‘United States Medical Licensing Examination (USMLE) Content Outline’ [22,23]. A six-member delegation from the taskforce was selected to visit a medical school with a curriculum that best aligned with NYU GLISOM goals. The delegation directly engaged with students, educational deans, and key faculty, and observed classes and workshops to gain insights into their curricular blueprint.

The taskforce undertook a deliberate screening process to select Course Directors based on their educational leadership experience and aligned with their clinical and academic expertise to lead specific workgroups for curriculum development. The task force was supported by employed external consultants to guide the process of curriculum innovation. Members of the expanded taskforce were then selected to establish the Evolving Physician Education Committee (EPEC) as the main curriculum committee whose scope of work included continuing development and design of curricular policies in addition to monitoring and evaluation of educational outcomes. The mission guided the overall educational program objectives.

### Establish curricular pillars and longitudinal courses

EPEC identified Basic, Clinical and Health Systems Sciences as the three curricular pillars of the school. The design of the accelerated curriculum provides for 48 weeks of pre-clinical learning during Year 1, focused on basic science and organ system principles. Years 2 and 3 focus on clinical experiences. The longitudinal courses of Clinical Ambulatory Practice Experience (CAPE), Health System Science (HSS), and Learning Community – Social Sciences, Humanities, Ethics and Professionalism (LC-SHEP) infuse holistic principles of primary care across all 3 years.

Key pedagogical approaches adopted were: 1) Early placement of experiential learning of primary care practice in CAPE; 2) Introduction of HSS from day one and integration throughout all 3 years of the curriculum; 3) Skill development in moral self-awareness, self-reflection, moral reasoning, and ethical analysis in LC-SHEP; and 4) Utilization of active learning through Problem-Based Learning (PBL) (Table 1) [24,25]. During the first year, students spend two afternoons per week in their Longitudinal courses (HSS, LC-SHEP and CAPE), and one afternoon per week in the second year. For the third year, they are immersed in a four-week longitudinal block experience. CAPE provides direct patient care experience monthly in year one and two (10 sessions) and one month block experience in year three.

**Table 1.** Problem-based learning and longitudinal course director interviews.

Longitudinal Courses & PBL	Guiding Principle	Primary Key Driver
**Continuity Ambulatory Practice Experience Course (CAPE)** is a longitudinal ambulatory experience in the primary care setting with the goal of transitioning students from being observers to direct providers of patient care. The students practice their skillsets by taking patient histories, performing physical exams and assessments, and clinical management.	CAPE provides a patient- and learner-centered curriculum, emphasizing longitudinal patient care in the primary care setting. CAPE provides learners with a clinical setting where the principles of the practice of medicine, health system science, organ systems, basic sciences, ethics, and humanities can be put into practice.	One key driver was the development of measurable learning objectives (LO) in the clinical setting. Development of a CAPE passport outlined learning objectives and catalogued achievement of milestones (Appendix 1). For example, faculty look for opportunities in the clinical setting to cover the learning objectives focused on patient communication about vaccines reviewed in the Year 1 basic science and organ system courses. Thus, the CAPE passport was designed to make learning more intentional.
**The Health Systems Science (HSS)** curriculum consists of three threads: I: HSS Core domains [26] Healthcare structures and processes Population, Public, and Social Determinants of Health. Clinical informatics and Health Information Technology, Value in Health care, Healthcare Policy & Economics Health System Improvement II: Evidence-based Medicine and Clinical Epidemiology III: Interprofessional Collaborative Care and Practice	The HSS curriculum’s overall objective is to develop a mindset for systems thinking [27]. System thinking is defined as an approach to problem-solving that recognizes and prioritizes the understanding of linkages, relationships, interactions and interdependencies among the components of the system that give rise to the system’s observed behavior [28]. We want our students to appreciate the dynamic nature and complexity of our healthcare system and how the various components interact to optimize patient outcomes. Year 1 focuses on building knowledge around HSS core domains whereas Years II and III focus on experiential learning utilizing capstone projects and integration of clinical experience to drive learning within the students’ practice environment	The primary key driver is the integration of HSS throughout the curriculum leveraging experiential learning. Students complete a capstone initiative in one of two tracks: quality improvement and patient safety or population health/clinical epidemiology research. We ensure alignment of the capstone to the student’s primary care residency track to nurture a system thinking mindset focused on primary care throughout their careers. One example of a capstone initiative is the improvement of screening for social determinants of health by a student in the pediatric track.
**Learning Community - Social Sciences Humanities Ethics and Professionalism (LC-SHEP)** prepares students to negotiate the complexities of clinical care and medicine in society. The four content domains of LC-SHEP: psychology of decision-making, history of medicine, systemic racism, and professional identity formation appear as longitudinal threads.	We worked deliberately to make the non-bio scientific and less tangible parts of medicine (social sciences, humanities, and ethics) into an outcomes-based curriculum. This ensures that the material reflected in the learning objectives is real, relevant, and integrated into the everyday work of our students as physicians. Content from a variety of disciplines within social sciences and humanities are woven together along with active skill development in moral self-awareness, self-reflection, moral reasoning, and ethical analysis.	We deeply integrated principles of sociology, cultural anthropology, humanities, ethics, and professionalism into students learning. These content areas were woven into longitudinal strands within a spiral curricular structure. For example, discussions of shaping the organization of allopathic medicine, definition of codes of ethics and medical professionalism, and ways in which elitism, bias, and racism have caused harm in LC-SHEP allow patient encounters with their primary care doctors to be viewed within these social contexts.
**The Problem Based Learning (PBL)** curriculum consists of 65 cases across Years 1–3. Students are evaluated and receive feedback on their group and individual PBL skills.	The overarching principle that guided PBL curriculum development was creating independent, self-directed, life-long learners and nurturing core clinical skills from day one of medical school. PBL has allowed us to reap the benefits of this learner centered approach to promote both knowledge goals (independent identification of knowledge gaps and analysis and synthesis of relevant information) and process goals (clinical reasoning, interprofessional collaboration, and critical, appraisal of literature).	PBL cases are adapted from real life clinical scenarios, which students will see in their day-to-day practice as primary care physicians. HSS LOs embedded in each case help define the unique role of the primary care physician-family partnership in provision of integrated, comprehensive healthcare delivery. This is critical to nurturing holistic primary care physicians.

Medical students are provided 1:1 clinical faculty mentorship, which are matched by students who selected primary care track from onset. The mentors were nominated by Department Chairs and approved by EPEC. In addition, for HSS capstone projects, students are assigned 1:1 project mentor from faculty who have expertise in quality improvement science and population health and clinical epidemiology research.

### Development of course goals and learning objectives

The curricular plan for the 3 years was developed to ensure that direct linkages were present in a waterfall-like cascade from the educational program objectives to the individual course goals. From here, individual session learning objectives were defined to guide the development of educational content. Further downstream on the waterfall cascade, each of the summative assessments was linked to the learning objectives and feedback loops were examined to maintain alignment with overall educational program objectives. A sub-group of EPEC worked closely with the course directors to make sure that the above linkages remained alive in the final materials.

### Horizontal and vertical integration

Mapping of learning objectives to early, mid, and late curricular years was undertaken to distribute LOs vertically and ensure appropriate progression across 3 years. Longitudinal course directors then met with the basic science thread leaders, organ systems directors, and clerkship directors to horizontally integrate learning objectives within each block. Iterative small group sessions using a ‘speed dating’ framework was employed, to initiate the concept and ideation of curricular design to optimize integration.

### Session design and instructional methods

Our major active learning approach centers on PBL. PBL cases were adapted from real life clinical scenarios, integrating learning objectives from HSS in every case. Small group discussion, workshop, Case or Team-based learning, research projects, and reflection were also employed in HSS and LC- SHEP.

CAPE was created based on the foundation of primary care. With this, students are matched with primary care discipline-specific clinical mentors. CAPE’s clinical experience is guided by specific learning objectives to drive integration with other longitudinal courses. After each clinical session, a Clinical Documentation Review is completed, which links the clinical encounter with one or more LOs and allows room for feedback.

Instructional methods for each session were coded with standardized nomenclature from MedBiquitous to ensure balance and diversity of instructional design [29].

### Curriculum mapping

Longitudinal course themes were defined based on LCME guidelines for a graduating medical student. Additional themes were added that were essential to our curricular mission. Themes included key content areas from the longitudinal courses including HSS and Integrated Population Health topics, primary care delivery processes, and global health and ethics. The themes included identified top societal issues based on the Community Needs Assessment data for the population served by our school.

We created a database using Curriculum Explorer and ExamSoft as tools to tag the courses and learning objectives, instructional methodology, and assessments with the longitudinal themes across the entire curriculum (Appendix 1). A dashboard was developed to easily identify core contents, gaps, and redundancy.

### Assessment

The Assessment sub-committee of EPEC was formed to be the central structure for promoting assessment and integration. This committee provided leadership, oversight, and coordination of assessment activities and periodic reviews of educational goals. A needs assessment of the faculty regarding assessment practices was conducted.

We conducted a faculty development workshop focused on the formulation of examination questions. Item writing specialists from the National Board of Medical Examiners (NBME) were invited to guide our internal faculty members. Leveraging the expertise of our course faculty as content specialists, examination items were crafted in alignment with the designated learning objectives of the session. Prior to administration, each item underwent scrutiny by select members of the assessment committee to ensure an appropriate balance between questions assessing application and those focusing on knowledge retrieval. Multiple-choice question (MCQ) examinations are administered bi-weekly throughout Year 1 of the curriculum, with uniform distribution of questions among all students. Each item was analyzed using point biserial and discrimination index [30]. The longitudinal courses focused on assessing interpretation and application through essays and reflections in LC-SHEP, project-based experiential learning in HSS, and completion of primary care clinical skills through the CAPE passport (Appendix 2). PBL implemented a rubric to assess both knowledge and PBL/EPA skill acquisition. Timely and frequent formative assessments were adopted to help students identify strengths and areas for improvement early on.

Short essay questions were created for each PBL case to allow students to self-assess, reinforce, and integrate important concepts of each case. They were created by course directors based on PBL director and facilitator feedback of student group performance during each session and high yield topics of each case. They were released to all students biweekly in year 1 and monthly in year 2 to allow further consolidation and uniformity of key concepts.

During the Clinical Clerkship Year, the assessment approach shifted to Work Based Assessments (WBA) that focused on 13 EPAs. The EPA taskforce was expanded to engage diverse stakeholders from nursing, administration, and community members to develop milestones for each critical competency linked to the EPAs. Assessment during the clerkship years focused on clinical performance and application of health systems science principles in care delivery.

### Course evaluation and continuous quality improvement

The overarching goal of the evaluation process was to gather data, analyze it, and provide useful feedback to course directors, administrators, and relevant parties on the quality and effectiveness of the curriculum and to assist in making curricular enhancements. The evaluation was guided by the Joint Commission on Standards for Educational Evaluation, including standards for utility, feasibility, propriety, accuracy, and accountability. All curricular changes were presented to EPEC for final approval, while other changes within the educational program could be made based on the evaluation information.

The evaluation of an undergraduate medical education program aligns with LCME accreditation Standard 8, necessitating ongoing curriculum management, evaluation, and enhancement to uphold and improve program quality. Standard 8 mandates the use of various data sources, including outcome data, program completion data, and evaluations of courses and instructors. This comprehensive evaluation process aims to furnish timely feedback to stakeholders such as course directors and administrators, facilitating informed decisions regarding curriculum modifications. While final approval rests with EPEC, the evaluation process empowers course directors and administrators to enact operational improvements based on the gathered information. Guided by Joint Commission standards, the evaluation prioritizes utility, feasibility, propriety, accuracy, and accountability. It incorporates multiple internal sources such as student evaluations of courses and faculty, faculty self-assessment, and aggregate student performance data, along with external sources like standardized exam results (e.g., USMLE Step 1 and 2 data) and graduation questionnaires (e.g., AAMC Graduate Questionnaire). This evaluation spans both pre-clinical and clinical years, with Year 1 courses and clerkships reviewed annually and the entire program evaluated triennially post-inaugural class completion.

## Outcomes

### Residency match results

For NYU GLISOM class of 2022 (*N* = 20), 90% (*N* = 18) of students matched/stayed in Primary Care disciplines. For the class of 2023 (*N* = 25), 64% (*N* = 16) matched/stayed in Primary Care disciplines and an additional 4 students matched in Psychiatry. Overall, 76% of NYU GLISOM medical students (*N* = 45) matched/stayed in Primary Care disciplines to date. Our school considers general surgery residency as part of the primary care tract; however, excluding the general surgery residents, NYU GLISOM’s retention rate is 67%, which is still more than double the national average of 30% [31,32].

### Graduated medical student perspective

The questions and excerpts from graduate student interview responses are summarized below.

Question 1: How do you feel the curriculum prepared you for a residency in primary care and as a health system change agent?

**Table:** 

Family Medicine Resident	- “Clinical experience right from the get-go…paired with the same physician throughout.” - “The Health System Science program was helpful for teaching us how the health system works…Having this broader knowledge…I am better able to coach my patients through the system by modifying their plans of care to accommodate for barriers.” - “The curriculum prepared us well for what it means to be a leader in a health system and what opportunities there are for better care for patients.”
Internal Medicine Resident	- “The inclusion of the impact of sociocultural factors on access to care in our curriculum was essential to our learning and growth as primary care physicians.” - “Most of my colleagues in residency have received zero training on the issues of billing and insurance, but…I feel fortunate that my medical school curriculum included these.” - “I find myself thinking about health…in the context of the broader range of healthcare as a health system.” - “Excellent faculty and early integration allowed me to feel prepared to deliver primary care as a change agent.”
Obstetrics & Gynecology Resident	- “Our CAPE experience throughout all three years really allowed us to get more exposure in the primary care field.” - “Our HSS course equipped us with tools like the HSS patient safety matrix, which continues to help me evaluate problems within the hospital through my residency.”
Pediatric Resident	- “Longitudinal aspects of the curriculum through CAPE: frequent and early exposure to pediatric patients…with the same preceptor for all of my years” - “The Quality Improvement (QI) project prepared me to think through the methodology for improving primary care practices and developing a QI mindset. Every resident in my program will have to do a QI project, and it is great that I already know how to structure one and the lessons from this are already impacting my work.”

**Table:** 

Family Medicine Resident	- ‘Simulation experiences were phenomenal and emphasized the importance of being able to meet the patient where they are…with great feedback.’ - ‘My knowledge and comfort with formal quality improvement was much further along than many of my resident colleagues… a mindset that we can apply to the work we are doing daily.’ - The organ-system based model ‘allowed us to connect the dots much quicker…in learning physiology, anatomy, pharmacology, etc.’
Internal Medicine Resident	- ‘It was a formative experience to learn how to build trust, empathy, and relationships with your patients in every situation during our first year.’ - ‘I rely on many of the practical skillsets we learned in our LC-SHEP course…when I am having difficult conversations with patients and their families.’ - ‘I find myself leaps and bounds beyond my colleagues.’
Obstetrics & Gynecology Resident	- ‘Our medical ethics course [LC-SHEP] was excellent at helping us develop strategies for addressing the mental load we experience in the hospital and being able to identify things we are uncomfortable with.’ - PBL helped us ‘learn how to look up information and find pitfalls in [our] knowledge.’
Pediatric Resident	- ‘The content of the curriculum and the fast-paced rigger also helped prepare me for the challenging work and pace of residency.’ - ‘I really liked the PBL learning environment of breaking down and dissecting cases in a collaborative format.’ - ‘It was also helpful to have simulation sessions for leading a code or sepsis … compared to some of my co-residents, I feel more comfortable performing these.’ - ‘Practicing difficult conversations and delivering bad news in a safe environment helped me feel much more comfortable doing so in residency.’

**Table:** 

Family Medicine Resident	- ‘It is hard to think of an idea for what should be added or improved, as I feel more prepared for residency than my peers.’
Internal Medicine Resident	- ‘Medical education is a huge component of being an academic physician…I had wished that in my 3rd year I had more opportunities to teach.’
Obstetrics & Gynecology Resident	- ‘I think the program could flesh out more about what it means to have a primary care medical school on a campus that is very specialized.’
Pediatric Resident	- ‘I think having more experiences in clinics that serve underserved communities would be beneficial to further cultivating health system leaders.’

Question 2: In your opinion, what were the main curricular drivers that positioned you best for residency?

Question 3: Name one aspect of the curriculum that could be improved or optimized to further support your development as a primary care physician.

All students interviewed stated that CAPE and HSS prepared them for residency in primary care and as a health system change agent. CAPE allowed early exposure to primary care patients. The longitudinal nature of the program allowed students to follow the same preceptor for all three years to build continuity of training and mentorship. The HSS curriculum allowed students to develop a system thinking mindset. Among longitudinal experiences, the main curricular drivers that students felt best equipped for residency included LC-SHEP, PBL, and HSS.

Aspects of the curriculum that could be optimized to further support development as primary care physicians included to offer more opportunities to develop students as teachers, for increased exposure to learn in advanced primary care settings, and to provide care to underserved communities. Notably, three of the four students interviewed felt ‘much further along’ and ‘more prepared for residency than their (my) peers’ in practicing within the healthcare system.

### Graduate Questionnaire (GQ) results

The GQ data for 2022 (*N* = 19) and 2023 (*N* = 23) revealed that 97.6% of NYU GLISOM students agreed or strongly agreed that they were satisfied with the quality of their medical education, and that it has done an excellent job of fostering or nurturing their development as a future physician and 93% for nurturing their development as a person (Appendix 3). This is in line with other accelerated pathway programs [33].

For the 2022 GQ data, NYU GLISOM performed above the 90th percentile for twelve domains deemed related to the longitudinal courses. For the 2023 GQ data, five of the domains were sustained above the 90^th^ percentile while seven of the twelve domains were rated within the 50–75^th^ percentiles.

### Residency readiness survey

Residency program directors provided information about 15 of the 20 students in our first graduating class in the AAMC Resident Readiness Survey [34]. In response to the question ‘During the transition to GME (0–6 months of PGY-1 year), did this resident meet overall performance expectations?’, all 15, 100% of our graduates met or exceeded expectations. This is compared with the national average of 96.9% (*n* = 14,347) who met or exceeded expectations.

### Graduate Questionnaire (GQ) results

The GQ data for 2022 (*N* = 19) and 2023 (*N* = 23) revealed that 97.6% of NYU GLISOM students agreed or strongly agreed that they were satisfied with the quality of their medical education, and that it has done an excellent job of fostering or nurturing their development as a future physician (Appendix 3).

For the 2022 GQ data, NYU GLISOM performed above the 90th percentile for twelve domains deemed related to the longitudinal courses. For the 2023 GQ data, five of the domains were sustained above the 90^th^ percentile while seven of the twelve domains were rated within the 50–75^th^ percentiles.

### USMLE results

For the class of 2022, 100% (*N* = 20) of students passed Step 1 and Step 2 on their first attempt. The Step 1 average score is at national average of 233 (*N* = 20). For Step 2, the NYU GLISOM score was 249 (*N* = 20), above the national average of 245.

For the class of 2023, 100% (*N* = 25) of students passed Step 1 on first attempt (new system Pass/Fail). For Step 2, 24 of 25 students (95%) passed on the first attempt, and one student passed on the second attempt. The average score was 250 (*N* = 25), above the national average of 246.

### HSS NBME results

For the class of 2022 and 2023 (*N* = 41), the HSS NBME mean score at the end of year three was 83.1 (*N* = 19) and 79.5 (*N* = 22), respectively, compared to the mean score of 69.9 (*N* = 437) using the NBME 2021–2022 national comparison group. 44% (*N* = 41) achieved 90%ile or above.

In addition, to assess the impact of the three- year curriculum, we administered the HSS NBME on entry for the 2022 class. The mean increased from 66 (on entry) to 83.1 (at end of instruction) and the percentile improved from 15.7% to 83.3%, respectively. This is a significant improvement from baseline following HSS instruction.

### Curriculum development workgroup perspective

A Strengths, Weaknesses, Opportunities and Threats (SWOT) analysis was completed by the curriculum development workgroup. During the brainstorming session, each member of the team was asked to write three strengths, weaknesses, opportunities, and threats individually and ideas were organized and summarized into broad themes (see Table 2).

**Table 2.** Strengths, weaknesses, opportunities and threats analysis.

Strengths	Weaknesses	Opportunities	Threats
Intentional design to support student primary care interest	Speed of Learning/accelerated program	Collaboration across the system	Regulatory Changes in GME
Integration of longitudinal course and PBL	Limited exposure to underserved communities	Strengthening local Primary Care Workforce and residency program	Increasing complexity of the patient population requiring redesign of primary care
Tuition Free	Current health systems focus on tertiary versus primary care	Faculty Development in population-based cares and health systems science	Increasing faculty and healthcare provider burnout

## Discussion

Our initial outcomes indicate that we have successfully operationalized an accelerated, standalone, integrated medical school curriculum mission-centered on primary care and health system leadership.

Our preliminary outcomes reveal a higher percentage of NYU GLISOM students entering primary care compared to national benchmarks. NYU GLISOM’s retention rate is 76%, which is more than double the national average of 30% [31,32]. Our school defines the primary care physician as a specifically trained physician providing first-contact care, taking continuing responsibility for the patient’s care, and dealing with all health problems [35].

Previous studies have raised concerns that accelerated programs may compromise student competency and readiness for residency [36,37]. However, our initial data from multiple sources including USMLE, HSS NBME, GQ, and RRS support that our students achieved national benchmarks for competency and readiness for residency in primary care, despite the accelerated curriculum. Graduate medical student perspective from focused interviews further substantiates our outcomes.

The 3-year integration of the longitudinal courses of HSS, LC-SHEP, and CAPE is a key pillar to reinforce the tenants of primary care and health system leadership. Despite the challenges of an accelerated program, NYU GLISOM successfully integrated the longitudinal courses with optimal performance and achievement of education program objectives. Our findings are in line with published literature on the value of longitudinal, multifaceted, primary care programs to increase the proportion of students choosing primary care specialties. Pfarrwaller et al. showed that programs with longitudinal primary care courses, primary care preceptorship, and project experience focused on primary care increase retention in primary care [31].

The strengths of this 3-year integrated primary care curriculum centered on the intentional design to support students’ specific primary care residency interests. The students declare their Primary Care residency interest from entry and are matched with faculty mentors from the same disciplines, similar to other schools’ programs [38,39]. The integration of the longitudinal courses and PBL are unique to our curriculum. The longitudinal courses of HSS and CAPE align their experiential learning experience to their chosen primary care track. The tuition-free nature of the school aligns with literature that supports ‘while debt does not seem to be the strongest predictor of specialty choice, it does have an impact on the career paths.’ [6–8,40,41] Overall, the tuition-free model’s impact on the primary care physician shortage remains to be seen but optimistically viewed as a driver to primary care retention.

The threats identified by faculty include no long-term regulatory funding solution to support primary care residency program expansion which could have UME downstream effects [42]. The increasing prevalence of chronic disease requires redesign of models of primary care systems to more team‐based care, population management approach. However, in most institutions, these approaches are in their early stages [43]. ‘Return on investment and the slower than anticipated rate in moving from fee‐for‐service to value‐based payment’ are challenges health systems face in redesigning primary care [43]. The increasing faculty and healthcare provider burnout that could lead to a drop in meaningful engagement with students is consistent with published literature on US medical school faculty burnout [44–46]. The weaknesses identified were the speed of knowledge acquisition necessary for an accelerated program, but as per our preliminary outcomes, our students successfully met national averages. Limited exposure to underserved communities, and the current health system’s focus on tertiary versus primary care. Presented a real opportunity to collaborate with other sites within the health system to improve exposure to diverse patient populations and clinical settings, strengthening the local primary care workforce and residency program, and to support ongoing faculty development in population-based care and health system science.

In addition, the longitudinal and PBL course directors identified faculty capacity and capabilities as main challenges to implementation of curriculum Maintaining faculty to student ratios for small group discussions and workshops and 1:1 mentorship entails the need to maintain sufficient numbers of highly trained faculty. LC-SHEP and HSS require training of faculty on new skill sets and disciplines, while CAPE and PBL require highly coordinated communication and coordination of faculty. Strategies employed include careful planning, ongoing discussions by the course directors, case writers, HSS capstone mentors, and LC-SHEP and PBL facilitators, and continuous quality improvements (CQIs). CAPE leveraged blast e-mails to communicate with faculty preceptors at diverse sites. HSS tapped leaders within the health system that may not be viewed as traditional medical school faculty, such as healthcare administrators and legislators.

Limitations of this study include small class size and limited data currently available from graduated students at a new school. Continued longitudinal studies of newly graduated classes will be helpful to investigate the sustainability of our model over time. Generalizability to other medical school systems may be impacted by smaller class size. Small class size may influence student success compared to schools with larger student numbers [47]. In addition, this feature article represents the NYU GLISOM faculty’s perspective about the curriculum which may have introduced bias in interpretation. Finally, there may be uncertainty of student’s continuous commitment to primary care following residency. Continued longitudinal analyses of our alumni will be helpful in ascertaining the lifelong success of our program.

Overall, we have successfully operationalized an accelerated, standalone, integrated medical school curriculum mission-centered on primary care and health system leadership. Our experience can serve as a model for innovation and design of an accelerated 3-year primary care curriculum.

## Appendices

**Table:**

Longitudinal Themes & Tags			
Access to Care (ATC)	End of Life Care (ELC)	Human Sexuality (SEX)	Opioid Epidemic (OPI)
Acute Care (ACC)	Ethics (ETH)	Identifying and Providing Solutions for Health Disparities (SOL)	Pain Management (PMG)
Adolescent Medicine (ADM)	Evidence Based Medicine (EBM)	Introduction to Clinical Medicine to the Patient (ICX)	Palliative Care (PAL)
Aging Population (AGP)	Genetics (GEX)	Law and Medicine (LAW)	Problem Based Learning (PBL)
Biomedical Informatics (BII)	Global Health Issues (GHI)	Medical Socioeconomics (MSF)	Radiology (RDG)
Chronic Care (CHC)	Healthcare Delivery Structure and Processes (HDP)	Medication Management and Compliance (MMC)	Rehabilitative Care (RHB)
Complementary Alternative (CAM)	Healthcare Policy (HCP)	Meeting the Healthcare Needs of Medically Underserved Populations (MUP)	Scientific and Ethical Principles of Translational Research (ETR)
Continuity of Care/Primary Care (CPC)	Healthcare Quality and Effectiveness (HQE)	Nutrition (NUT)	Scientific Methods (SCM)
Cultural Competence (CCP)	Health Promotion/Wellness (HPW)	Obesity (OBS)	
Determinants of Health (DOH)	Human Development/Life Cycle (HDV)	Oncology (ONC)	

## Appendix 2: Sample of Continuity Ambulatory Practice Experience (CAPE) Passport

**NYU GLISOM Continuity Ambulatory Practice Experience (CAPE) Passport**

**Block Two:**

**1**. **History**

□ OBTAIN a social history with a focus on substance use

□ DESCRIBE the 8 core communication behaviors that facilitate gathering data during the Medical Interview

□ DEFINE the structure and function of the Medical Interview

□ REFLECT on your current level of clinical skills as defined by the RIME assessment tool

**2**. **Communication**

□ RECALL the core role of the Medical Interview in the Doctor–Patient Relationship

□ DEMONSTRATE 1 or more of the 6 core communication skills that facilitate the Doctor–Patient relationship during a patient encounter

□ IDENTIFY the key gender, cultural and ethical issues when communicating with patients, their families and care givers

**3**. **Clinical Skills**

□ PERFORM medication reconciliation, REFLECT on its importance with compliance and adherence

□ DISCUSS with your preceptor, a new Rx and strategies to ensure patient understanding (Ex. teach back techniques)

**4**. **Screening, prevention and socio-structural integration**

□ PRESENT a screening measure using a patient and the USPSTF Prevention Task Force app or US preventive Services Taskforce website

□ RECOGNIZE and REFLECT on the communication needs of various populations (e.g., ESL, ASL, Braille, other spoken languages)

□ DEMONSTRATE the ability to effectively identify, activate and coordinate resources within the health system and work collaboratively and effectively across disciplines to advocate for patients (Ex. homecare planning, prior authorization, radiologic services, formulary issues within issuers)

□ DESCRIBE an understanding of ways in which integration of multiple disciplines is necessary for high value care

**5**. **Health System Science**

□ IDENTIFY one community health partner that your practice uses to refer patients to and the service they provide

□ DESCRIBE 2 factors that can contribute to medication non-adherence in chronic illness and identify strategies to mitigate

□ DISCUSS one primary or secondary prevention strategy used in your CAPE practice and EXPLAIN its health impact on the patient and population (Healthy people 2030; colon cancer screening)

□ DEFINE co-pay and deductibles. Explain how co-pay can vary for different services within the same plan (Hint: talk to the practice manager or the equivalent)

## Appendix 3: GQ Data

**Table:**

GQ Domain	NYU GLISOM 2022 (50–90% Benchmark, *N* = 152 Schools)	NYU GLISOM 2023 (50–90% Benchmark, *N* = 153 Schools)
Overall, I am satisfied with the quality of my medical education	100.0** (90.4–95.7)	95.5* (89.8–95.7)
Biostatistics & Epidemiology (Longitudinal Course Domain)	89.5** (71.0–85.3)	72.7 (74.1–85.3)
Behavior Science (Longitudinal Course Domain)	100.0** (89.3–94.4)	95.5* (91.0–95.7)
I have the communication skills necessary to interact with patients and health professionals.	100.0** (98.6–100)	100.0** (98.7–100)
I have basic skills in clinical decision making and the application of evidence-based information to medical practice.	100.0** (95.5–98.6)	100.0** (96.3–98.8)
I have a fundamental understanding of the issues in the social sciences of medicine (e.g., ethics, humanism, professionalism, organization and structure of the health care system).	100.0** (95.1–98.6)	95.5 (95.8–98.7)
I understand the ethical and professional values that are expected of the profession.	100.0** (98.1–100)	100.0** (98.3–100)
I believe I am adequately prepared to care for patients from different backgrounds.	100.0** (95.2–99.0)	100.0** (96.5–99.0)
I have the skills to apply the principles of high value care (e.g., quality, safety, cost) in medical decision-making.	100.0** (83–91.2)	90.9* (85.9–92.3)
I have the skills to address the social determinants that differentially influence the health status of patients.	100.0** (90.3–96.4)	95.5* (92.2–96.5)
My medical school has done a good job of fostering and nurturing my development as a person.	100.0** (70.5–83.6)	86.4** (71.3–83.9)
My medical school has done a good job of fostering and nurturing my development as a physician.	100.0** (92.1–96.8)	95.5* (92.4–97)

**Indicates above 90^th^ percentile; *Indicates above 75^th^ percentile.
